# AFLP reveals low genetic diversity of the bryozoan *Pectinatella magnifica* (Leidy, 1851) in the Czech Republic

**DOI:** 10.1186/s40709-017-0069-8

**Published:** 2017-11-25

**Authors:** Vendula Moravcová, Jana Moravcová, Vladislav Čurn, Zuzana Balounová, Josef Rajchard, Lenka Havlíčková

**Affiliations:** 1 Faculty of Agriculture, University of South Bohemia, 370 05 České Budějovice, Czech Republic; 2 Department of Biology, University of York, York, YO10 5DD UK

**Keywords:** Invasive species, Non-native species, *Pectinatella magnifica*, Bryozoan

## Abstract

**Background:**

Non-native species have aroused scientific interest because of their ability to successfully colonise areas to which they have been introduced, despite their sometimes limited genetic variation compared to their native range. These species establish themselves with the aid of some pre-existing features favouring them in the new environment. *Pectinatella magnifica* (Leidy, 1851), the freshwater magnificent bryozoan, is non-native in Europe and Asia. This study was designed to determine the genetic diversity and population structure of *P. magnifica* colonies collected from the Protected Landscape Area (PLA) and UNESCO Biosphere Reserve Třeboňsko (the Czech Republic) in the 2009 and 2011–2014 periods using Amplified Fragment Length Polymorphism (AFLP).

**Findings:**

The vast majority of the examined non-native colonies, except three colonies sampled in 2012, expressed very low levels of genetic variation, not differentiating from the USA native colony. The Bayesian clustering approach grouped the 28 accessions into two genetically different populations.

**Conclusions:**

The data suggest relatively low gene diversity within all colonies, which might reflect the recent expansion of *P. magnifica* in the Czech Republic.

**Electronic supplementary material:**

The online version of this article (10.1186/s40709-017-0069-8) contains supplementary material, which is available to authorized users.

## Findings

### Background

*Pectinatella magnifica* (Leidy, 1851), a freshwater bryozoan species that naturally inhabits lakes and rivers in eastern North America [1, 2], has been recently spreading in Europe and western Asia [3–5]. Its colonies begin life from a germinated statoblast (a product of asexual reproduction) or settled larva (the result of sexual reproduction). Once formed, colonies exude a protective gelatin-like matrix of exopolymers, enabling attachment to different submerged natural or artificial substrates [6] as flat sheets (in the form of thin films), as sessile globular colonies, or as gelatinous balls floating in water. The colonies are covered on their surface with rosettes, each containing 12–18 genetically identical zooids and can lead to considerable dimension and weight, as in the case of nearly 1 m diameter and 70 kg colony described by Balounová et al. [7]. Recent studies have focused on the determination of evolutionary relationships within *P. magnifica*, incorporating morphological traits (such as the morphology of the statoblasts, white spots on lophophora, pigmented mouth), molecular approaches, or a combination of both morphological traits and genetic data [8]. Most systematic (phylogenetic) studies including *P. magnifica* have used nuclear ribosomal (*ssrDNA, lsrDNA*) and mitochondrial (*rrnL, rrnS, cox1, cox3, cytb*) genes [9–13]. However, to determine intraspecific relatedness, ribosomal and mitochondrial genes are not sufficient, as demonstrated for instance in the case of using mitochondrial cytochrome c oxidase subunit I gene (*cox1*) for intraspecific analysis of geographically distinct colonies of *P. magnifica* in Korea [8]. AFLP markers are considered as one of the most popular tools for genetic analysis in the fields of evolutionary genetics, ecology and conservation of genetic resources [14]. The technique combines a high-information content and fidelity with the possibility of carrying out genome wide scans. However, a potential problem with this technique is the lack of homology of bands with the same electrophoretic mobility, what is known as fragment-size homoplasy [15]. This study is the first of its kind, where the random whole genome AFLP analysis was applied to determine relatedness of geographically and time-distant colonies of *P. magnifica.* The results provide the first comprehensive summary of the methodology used.

### Methods

**Fig. 1:**
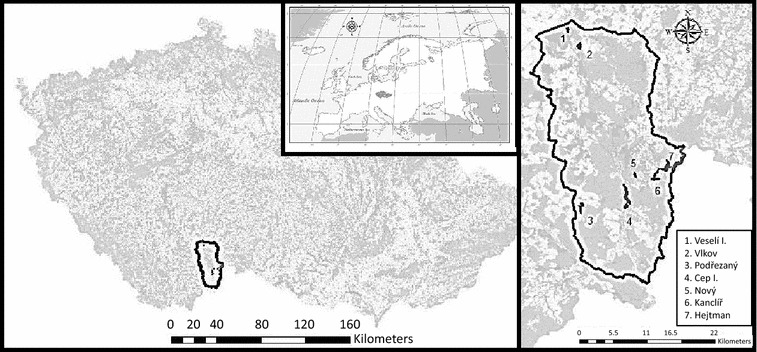
Map representing the sampled locations from the Třeboňsko region in the Czech Republic

A total of 29 samples from colonies of *Pectinatella magnifica* were collected between July and September in 2009 and 2011–2014. From these, 28 colonies were sampled from seven different locations within the Protected Landscape Area and Biosphere Reservation of Třeboňsko (in the Czech Republic) (Fig. 1) and one representative sample was collected from the native region of *P. magnifica*, in the Pacific Northwest of the USA (Additional file 1: Table S1). For each geographic location, up to eight transects of 20 m^2^ (10 m along the coast by 2 m into the water) were used to search for and sample statoblasts from colonies. The samples were fixed in 96% ethanol and stored at 4 °C, or were directly stored without fixation in − 20 °C.

Total genomic DNA was extracted from statoblasts by using DNeasy^®^ Blood & Tissue kit (Qiagen, Valencia, CA, USA) according to the manufacturer’s protocol. The quality and quantity of obtained DNA was verified by using a spectrophotometer BioSpec Nano (Shimadzu, Tokyo, Japan). The DNA was tested by using specific primers (Pm28SF: 5′-CTTTTCAGCCAAGCACATGA-3′ and Pm28SR: 5′-AAACCTCGCGTAGAGCAAAA-3′), designed for this study with Primer3 software [16], amplifying a fragment of 398 bp. Primers were designed based on the unique *P. magnifica* 28S rRNA gene sequence available in GenBank (FJ409576.1), and were chosen on the basis of their performance and specificity against alternative primers. The specificity was confirmed by sequencing. PCR was performed with 3 ng of DNA template, mixed with 1 µl (10 µM) of each primer, 12.5 µl PPP MM (TopBio, Prague, Czech Republic) and distilled water to make a final volume of 20 µl. PCR was performed using the following programme: pre-denaturation for 5 min at 95 °C, 35 cycles of 30 s at 95 °C, 1 min 60 °C and 40 s at 72 °C, finally, 10 min at 72 °C. PCR products were subjected to electrophoresis on 1.5% agarose gel in 1 × TBE buffer, and detected through ethidium bromide staining.

AFLP analysis was carried out as described by Vos et al. [17] with four primer combinations: fluorescent dye-labelled primer *Eco*RI + ACG/*Mse*I + AGT, *Eco*RI + ACG/*Mse*I + ACC, *Eco*RI + ACG/*Mse*I + AAC and *Eco*RI + ACG/*Mse*I + ATT.

One microliter of selective PCR amplicons, 0.4 μl of the GeneScan™ 500 LIZ^®^ (Life Technologies, USA) and 11 μl of formamide, were denatured at 95 °C for 5 min, cooled on ice immediately and directly forwarded to an capillary electrophoresis on an ABI 3500 genetic analyzer (Life Technologies, USA) and data were analysed by GeneMapper 5.0 software (Life Technologies, USA). Signals obtained from fragment analyzer were transformed into a binary character matrix with 1 for the presence or 0 for the absence of a band at a particular position.

Cluster and principal component analysis (PCA) were performed. These analyses were calculated by using R software (cran.r-project.org, version 3.2.3, accessed in 2015). Analysis of the genetic structure of the population was carried out assigning samples to *K* populations according to allele frequencies of each locus by using Bayesian model-based clustering method with Structure 2.3.4 [18]. The program was run 10 times at each of 10 different *K* values (1–10) with a period of 5000 burn-in steps followed by 50,000 iterations. The admixture model with independent allele frequencies was used in all analyses. The number of populations was inferred by plotting the log probability of the data [Ln P(D)] for each *K* value. Also, in order to determine the most appropriate number of *K*, Δ*K* was calculated as described by Evanno et al. [19].

### Results

AFLP analysis with four primer combinations amplified 496 bands, ranging in size of 36–480 bp, from which 79.4% were polymorphic (394 bands). The number of polymorphic fragments for primer combination ranged from 72 to 132 (Table 1), with a mean of 180 ± 5 (SE) total bands obtained from all primer combinations (125–225) per accession.

**Table 1:** Restriction enzyme primer combinations from Amplified Fragment Polymorphism

AFLP selective primer extensions	Band size	No. polymorphic bands	Polymorphic
E-ACG/M-AAC	44–480	103	79.8%
E-ACG/M-ACC	43–301	72	73.5%
E-ACG/M-AGT	36–414	132	84.1%
E-ACG/M-ATT	43–399	87	77.7%
Total		394	79.4%

**Fig. 2:**
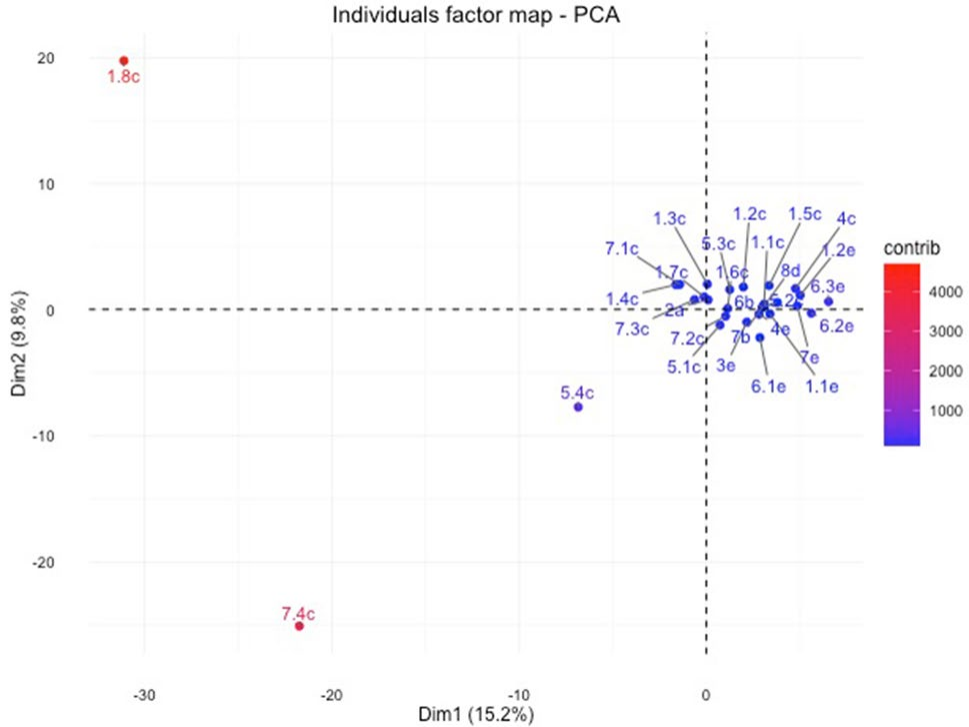
Principal Component Analysis (PCA) identifies two directions, Dim1 (PC1) and Dim2 (PC2) along which the data have the largest spread. Samples are coloured according to their contribution for a given observation (blue, low contribution; red, high contribution). Samples were given numbers according to location: Veselí I (1), Vlkov (2), Podřezaný(3), Cep I (4), Nový Kanclíř (5), Hejtman (6), Staňkovský (7), USA (8) and they were given letters according to the year of collection: 2009 (a), 2011 (b), 2012 (c), 2013 (d) and 2014 (e)

**Fig. 3:**
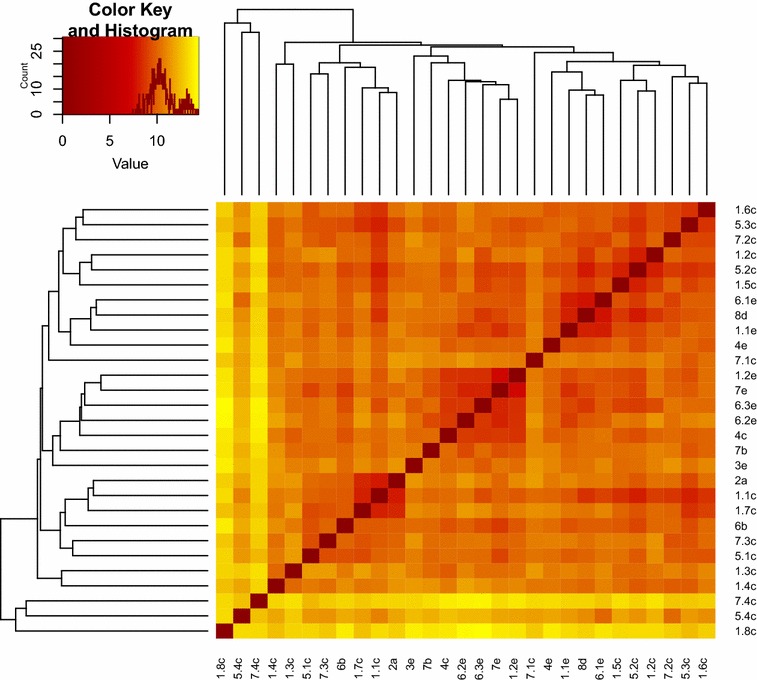
Heat map with dendrogram for visualizing distances between 29 samples of *P. magnifica* according to AFLP analysis. The colour coding is proportional to the value of the dissimilarity between observations: dark red if dist(xi,xj) = 0 and bright yellow if dist(xi,xj) = 1. Sampled locations in the Czech Republic: Veselí I (1), Vlkov (2), Podřezaný (3), Cep I (4), Nový Kanclíř (5), Hejtman (6), Staňkovský (7), and USA (8); within years 2009 (a), 2011 (b), 2012 (c), 2013 (d) and 2014 (e)

Twenty-nine genotypes were analyzed by cluster analysis and principal component analysis (PCA) to define genetic distance within accessions. First and second principal component of PCA analysis explained 15.2 and 9.8% variation, respectively (Fig. 2) and together with distance matrix applying Euclidian argument, clearly distinguished three samples which showed highly different AFLP profile, namely: 1.8c, 7.4c, 5.4c (Fig. 3). The highest distance (14.39%) was observed between samples 1.8c and 6.3e, followed by 14.21% in samples 1.8c and 6.2e and 14.07% between samples 6.3e and 7.4c. The lowest distance was observed between samples 1.2e and 7e (7.42%), 8d and 6.1e (7.75%), and same distance of 7.94% was observed between samples 5.2c and 8d and also 2a and 1.1c.

The STRUCTURE results divided samples into two groups (*K* = 2) with no consistent trend differentiating individual localities or years of collection by using these groups (Additional file 2: Table S2).

### Discussion

Recent studies show that the level of expansion of the non-native *Pectinatella magnifica* is increasing not only in Europe and Asia, but also within the country of its native origin [2, 4, 5, 8, 20]. The AFLP technique is very popular in population and evolutionary genetic studies of organisms with no prior sequence knowledge, aiming to assess the degree of variability and genetic structure among tested individuals. The advantage of this technique is genome wide screening, which provides a high information content. However, every methodology can have challenges and in the case of AFLP, the evaluation of fragments can be misleading due to size migration, which may lead to assigning fragment lengths to loci of different origin in the genome [14].

In this study, the AFLP technique has been used to characterize the population structure of *P. magnifica*, with the aim to better understand the invasion pattern of this species. The results showed a low level of genetic diversity within all colonies sampled, with low differentiation between sampling locations. Also, STRUCTURE results support the hypothesis that a single metapopulation exists without any significant genetic differentiation. In the study of Kang and An [8], the comparison of 20 sequences of the *cox1* gene from *P. magnifica* colonies collected from three different watersheds in Korea did not show any variation between the colonies. Low genetic diversity was also observed in other plant and animal invaders, including species which utilize both, sexual and asexual reproduction [21–24]. The main reason for low genetic diversity within populations of non-native organisms in secondary areas of occurrence may be attributed to their recent establishment. Most likely, only a small fraction of the source germplasm may have been the initial contaminant, generating an extreme diversity bottleneck in the newly established population with weak or low population structure. The results indicate a recent invasion, where colonies likely originate from single or closely related sources. This fact clearly demonstrates that a high amount of genetic diversity is not necessarily a condition for global spread over contemporary timescales. Given the fact that *P. magnifica* was first reported in Europe in 1883 [7], a low diversity across Europe is expected, although it could also be possible that *P. magnifica* was previously undetected. The results of genetic analyses performed in this study on *P. magnifica* colonies sampled within 5 years across different localities correspond with the so called genetic paradox in invasive species, where invasive populations are able to overcome low genetic diversity and inbreeding to thrive in the invaded environment. During optimal conditions, colonies increase in size through rapid repeated asexual budding of new zooids, but sexual events also occur regularly and play an important role in creating genetic variation in progeny. However, based on the assumption of a low initial degree of total allelic variation in all colonizing individuals, rapid increase of genetic diversity cannot be expected through recombination events and it is not clear how important the amounts of genetic diversity are for persistence and spread in the future.

The genetic diversity between different transects and over the years was not very different, but showed a clustering pattern in term of years, where samples taken in the same year grouped more closely than the samples from the same locality collected within years. The USA sample representing the native genetic resource did not differ from other samples representing invasive genomes. This phenomenon could be explained as being a result of low general diversity between these genotypes but also as erroneous estimation of co-migrating bands in AFLP analysis.

In conclusion, our results showed the presence of a single metapopulation in all recently known localities across the Czech Republic with low or slight differentiation of *P. magnifica* colonies from the different locations or over the years respectively, and relatively low gene diversity. We suggest that the genetic pattern observed in this study might reflect the recent expansion of *P. magnifica* in PLA (during the last decades), and the colonization of new water streams, dams and ponds. In spite of its geographic distance, the sample from the native origin has shown no particular AFLP band pattern.

## Additional files

**Additional file 1: Table S1.** Detailed description of the samples location including GPS.

**Additional file 2: Table S2.** Detecting the number of clusters of individuals by using delta *K*.
